# Multimodality imaging to assess diagnosis and evaluate complications of large vessel arteritis

**DOI:** 10.1016/j.nuclcard.2024.101864

**Published:** 2024-04-24

**Authors:** Ayaz Aghayev, Brittany Weber, Tiago Lins de Carvalho, Andor W.J.M. Glaudemans, Pieter H. Nienhuis, Kornelis S.M. van der Geest, Riemer H.J.A. Slart

**Affiliations:** 1 Cardiovascular Imaging Program, Department of Radiology, Brigham and Women’s Hospital, Harvard Medical School, Boston, MA, USA; 2 Division of Cardiovascular Medicine, Department of Medicine, Brigham and Women’s Hospital, Harvard Medical School, Boston, MA, USA; 3 Medical Imaging Center, Department of Nuclear Medicine and Molecular Imaging, University Medical Center Groningen, University of Groningen, the Netherlands; 4 Department of Rheumatology and Clinical Immunology, University Medical Center Groningen, University of Groningen, the Netherlands; 5 Department of Biomedical Photonic Imaging, Faculty of Science and Technology, University of Twente, Enschede, the Netherlands

**Keywords:** Large vessel vasculitis, Multimodality imaging, PET, Complications, Future trends

## Abstract

Different types of vasculitis can be distinguished according to the blood vessel’s size that is preferentially affected: large-vessel, medium-vessel, and small-vessel vasculitides. Giant cell arteritis (GCA) and Takayasu’s arteritis (TAK) are the main forms of large-vessel vasculitis, and may lead to lumen narrowing. Clinical manifestations of arterial narrowing on the short- and long term include vision loss, stroke, limb ischemia, and heart failure. Imaging tools are well established diagnostic tests for large-vessel vasculitis and may aid therapy monitoring in selected cases while providing important information regarding the occurrence of vascular damage, tissue and organ complications. This review aims to provide the current status of multimodality imaging for the diagnosis and identification of vascular complications in the field of large vessel vasculitis.

## INTRODUCTION

The primary vasculitides are autoimmune diseases affecting blood vessels. Different types of vasculitis can be distinguished according to the blood vessel’s size that is preferentially affected: large-vessel, medium-vessel, and small-vessel vasculitides [1]. Giant cell arteritis (GCA) and Takayasu’s arteritis (TAK) are the main forms of large vessel vasculitis (LVV) [2,3].

GCA is prevalent among older individuals (>50), with the highest incidence noted in individuals of Northern-European descent. GCA may cause inflammation of both large systemic arteries (large vessel GCA; lvGCA) and medium-sized cranial arteries (cranial GCA; cGCA). The complications of cGCA include blindness and stroke, whereas aortic dissection is a feared complication of lvGCA [4]. A meta-analysis of studies with systematic imaging reported a pooled prevalence of thoracic aortic aneurysms of 15% [5]. On the other hand, TAK primarily occurs in young females, with a predilection for those of Asian descent. TAK more commonly causes focal stenotic lesions than GCA. Hence, loss of peripheral pulsations is more frequently observed in TAK than in GCA. In addition, TAK more frequently causes symptomatic stenotic lesions in the visceral arteries than GCA [6].

Comprehensive clinical and laboratory evaluation is critical in patients with vasculitis. For long, temporal artery biopsy was the standard test for GCA, while conventional angiography was the main test for TAK. However, other imaging methods have now largely replaced these tests. This includes ultrasonography (US), magnetic resonance angiography (MRA), computed tomography angiography (CTA), and [^18^F]-fluoro-2-deoxy-D-glucose positron emission tomography/computed tomography (FDG-PET/CT). These imaging tools are well established diagnostic tests for LVV. In addition, these imaging techniques may aid therapy monitoring in selected cases while providing important information regarding the occurrence of damage. Overall, these imaging modalities provide complementary information to each other and can therefore be used in parallel.

This review aims to provide the current status of multimodality imaging for the diagnosis and identification of vascular complications in the field of LVV.

## MULTIMODALITY IMAGING IN LARGE VESSEL VASCULITIS

**Figure 1.**
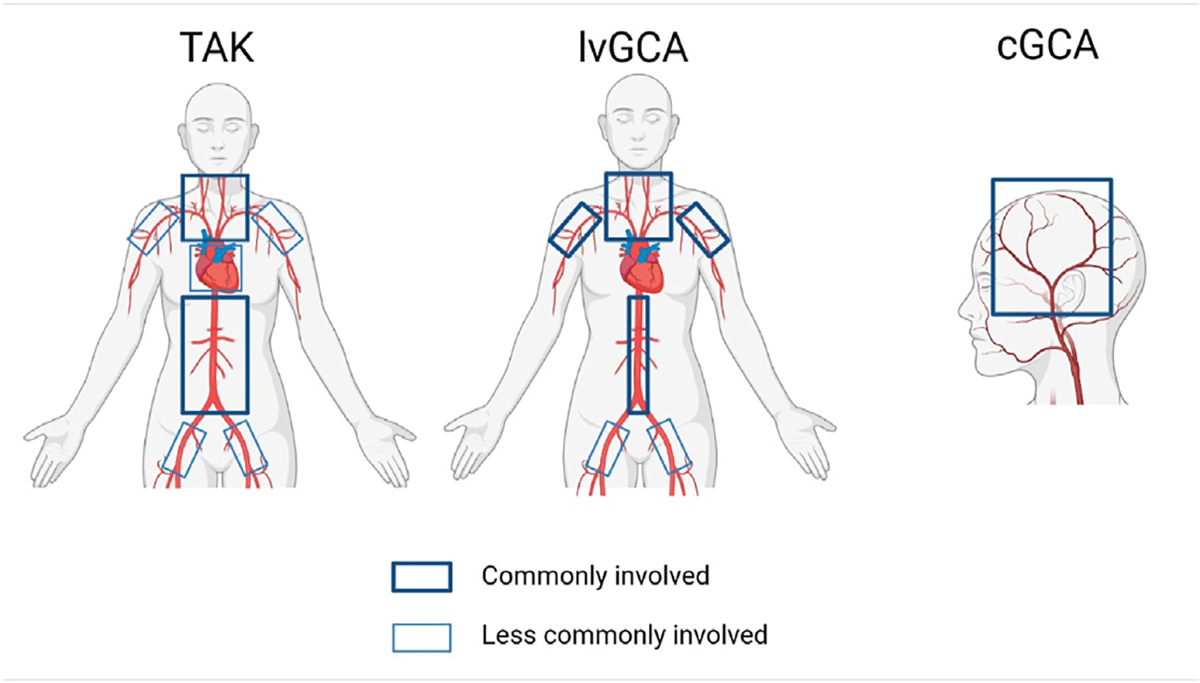
Disease classification and arterial involvement in large vessel vasculitis. Created with BioRender.com. Variation exists across the phenotypic spectrum of LVV, but patterns of arterial involvement may help to distinguish GCA from TAK. lvGCA more commonly affects the axillary arteries, whereas TAK is more likely to affect the renal and mesenteric vessels. Overlap between cranial cGCA and lvGCA is commonly found. LVV, large vessel vasculitis; GCA, giant cell arteritis; TAK, Takayasu’s arteritis; lvGCA, large vessel GCA; cGCA, cranial GCA.

Imaging plays an increasing role in the diagnosis and management of LVV. Although temporal artery biopsy is an established diagnostic test for cGCA, it has limited specificity and is of little use in patients with limited lvGCA, TAK or cGCA with isolated involvement of cranial arteries, such as vertebral arteries (see Figure 1). In cases of TAK or lvGCA, histology evidence is typically not possible. Such analysis is only possible in cases requiring an aortic replacement surgery, often many years after the diagnosis has been established by imaging. While traditional imaging methods encompass morphological evaluations through US combined with Color Doppler ultrasonography (US/CDUS), CTA, and MRA, FDG-PET/CT(A) is already a well-established imaging tool, and FDG-PET/MR(A) is gaining traction as an imaging tool for LVV. Catheter-based techniques are no longer central to LVV diagnosis due to their inability to inform on the vessel wall and invasiveness. Instead, they are now primarily used for therapeutic interventions. It is important to realize that the various imaging methods provide complementary information. US/CDUS, CTA, and MRA primarily reveal thickening of the arterial wall. The latter two may also reveal contrast enhancement of the arterial wall, whereas US/CDUS provides insight into the blood flow. In contrast, FDG-PET measures metabolic activity in the arterial wall. Although these are all established tools for the diagnosis of lvGCA, their role in therapy monitoring is less clear since imaging abnormalities may persist despite prolonged clinical remission. In selected cases, a combination of imaging tools is needed to gain insight into the inflammatory state of the vessel wall.

### Ultrasound with color doppler

US/CDUS is commonly used as the primary noninvasive method to diagnose LVV. The advantages are the lack of radiation exposure, the nonrequirement of nephrotoxic contrast agents, widespread availability in most centers, and cost-effectiveness. US/CDUS is the first-line imaging modality of choice in patients with suspected GCA. It can be utilized in the head and neck vasculature as well as in extremity arteries. Two hallmark US imaging signs indicative of cGCA can be observed. The first is the “halo sign” which appears as a hypoechoic, circumferential rim around the vessel lumen in the transverse view of the ultrasound, signifying inflammation-induced wall thickening and edema [7,8]. The threshold for the wall thickening is considered when an intima-media thickness of .29 to .42 mm in the distinct temporal artery segments and 1.0 mm for the axillary arteries [9]. The second is the “compression sign” A healthy vessel, when pressed with a US probe, collapses entirely, blending in with the adjacent subcutaneous fat. However, an inflamed vessel with a thickened wall remains visible even when compressed [10]. In TAK patients, US/CDUS can be used to assess abnormalities in the carotid, subclavian, axillary, and vertebral arteries. A classic ultrasound imaging finding in TAK is the “macaroni sign,” defined as circumferential echogenic thickening of the vessel wall [11]. Contrast-enhanced US is an additive technique in identifying vessel wall neovascularization, a marker for active inflammation, and has been effectively used to differentiate between active and inactive TAK [6].

In terms of diagnostic accuracy for cGCA, and US/CDUS has a sensitivity of 77% and a specificity of 96%, using temporal artery biopsy as a reference standard [12]. Studies have shown that the combination of clinical pretest probability scoring (by Southend GCA probability score; SGCAPS) and US/CDUS correctly classify the majority of patients with cGCA [13–15]. In TAK, the reported diagnostic accuracy shows a sensitivity of 81% and a specificity of above 90% [16]. US/CDUS is also a valuable tool for evaluating LVV complications, especially in the vasculature of the neck and extremities. While the US provides several benefits, its major disadvantages are the dependence on the skill of the operator/sonographer, and the lack of penetration of the deep or intra-thoracic vessels. Therefore, additional imaging methods are often required.

### Computed tomography angiography

CTA has high spatial resolution and can detect LVV and complications. It also has the ability to distinguish vasculitis from atherosclerosis or acuteaortic syndromes, such as intramural hematoma or thrombosed false lumen/dissection [17]. Classic imaging findings of LVV are circumferential thickening of the wall (exceeding 2–3 mm in the aorta) and wall enhancement on delayed image acquisition [18]. Hyper-enhancement of the outer vessel layers, including the adventitia and media, along with poor or hypo-enhancement of the intima due to swelling or edema, results in the “double ring” sign, commonly seen in TAK [19]. The diagnostic accuracy of CTA for idiopathic lvGCA is variable, with sensitivity rates ranging between 73% and 95%, and specificity between 78% and 100% [20–22]. Lastly, CTA is an ideal tool to detect complications associated with LVV, such as atherosclerosis/calcification, aneurysm, dissection, or luminal narrowing/stenosis [23]. In TAK patients presented with chest pain, ECG-gated cardiac CT can be performed to assess aortic root and coronary artery complications [24]. A disadvantage of CTA is the radiation risk, particularly pertinent for younger female TAK patients necessitating extended imaging surveillance. In addition, patients with a clinical presentation of polymyalgia rheumatica or fever of unknown origin, CTA is not a preferred imaging modality as PET/CT will be more informative for confirmation of polymyalgia rheumatica alternative causes of inflammation.

### Magnetic resonance angiography

MRA is an advanced imaging tool that offers high-contrast resolution and relatively better spatial resolution. Its major advantage is the lack of radiation exposure. Due to this benefit, it is the recommended imaging modality for long-term vasculitis monitoring, especially in younger patients. Specifically, the European League Against Rheumatism endorses MRA as the primary imaging modality for evaluation in suspected cases of TAK [25]. The imaging features of MRA are circumferential wall thickening and enhancement on post-contrast images [17]. The ideal sequence is the pre- and post-contrast T1-weighted black blood sequence with fat supression. Almost all of these sequences utilize a contrast agent. However, an alternative MRA technique, the time-of-flight (TOF) sequence, can be employed without contrast. Initial studies have shown that a hyperintense signal on T2-weighted images in the vessel can aid in detecting active inflammation; however, this is often due to artifacts and is not reliable [25]. Similar to CTA, MRA can detect complications associated with vasculitis, such as severe stenosis or aneurysmal dilatation. However, it is less effective in detecting calcifications. Post-processing techniques, such as maximum intensity projection (MIP) images, especially in TOF sequences, are helpful in delineating the extent of stenoses or aneurysms. However, these techniques can exaggerate the degree of stenosis, making it extremely important to review the source images as well. Given that LVV, particularly TAK, can involve the aortic valve, cine (steady-state-free-precession) sequences can be obtained to assess valvular abnormalities.

For lvGCA, MRA post-contrast imaging shows a diagnostic sensitivity of 79% and a specificity of 96%, using temporal artery biopsy as a reference [26]. A recent meta-analysis revealed that for TAK, MRA achieved both a diagnostic sensitivity and specificity rate of 92% [16]. However, more extensive studies on the accuracy of MRA in diagnosing LVV are lacking. Recently, a high-resolution MRI of the superficial cranial vasculature has been shown to be an effective tool for diagnosing cGCA [27]. Its diagnostic accuracy is comparable to US/CDUS in detecting mural contrast enhancement in affected superficial cranial arteries. This approach can complement body MRA, offering simultaneous assessments of cGCA and LVV. The primary drawbacks of MRA for vascular imaging are the typically longer image acquisition times and the higher associated costs.

### [18F]-fluoro-2-deoxy-d-glucose positron emission tomography/computed tomography(A)/magnetic resonance(A)

**Figure 2.**
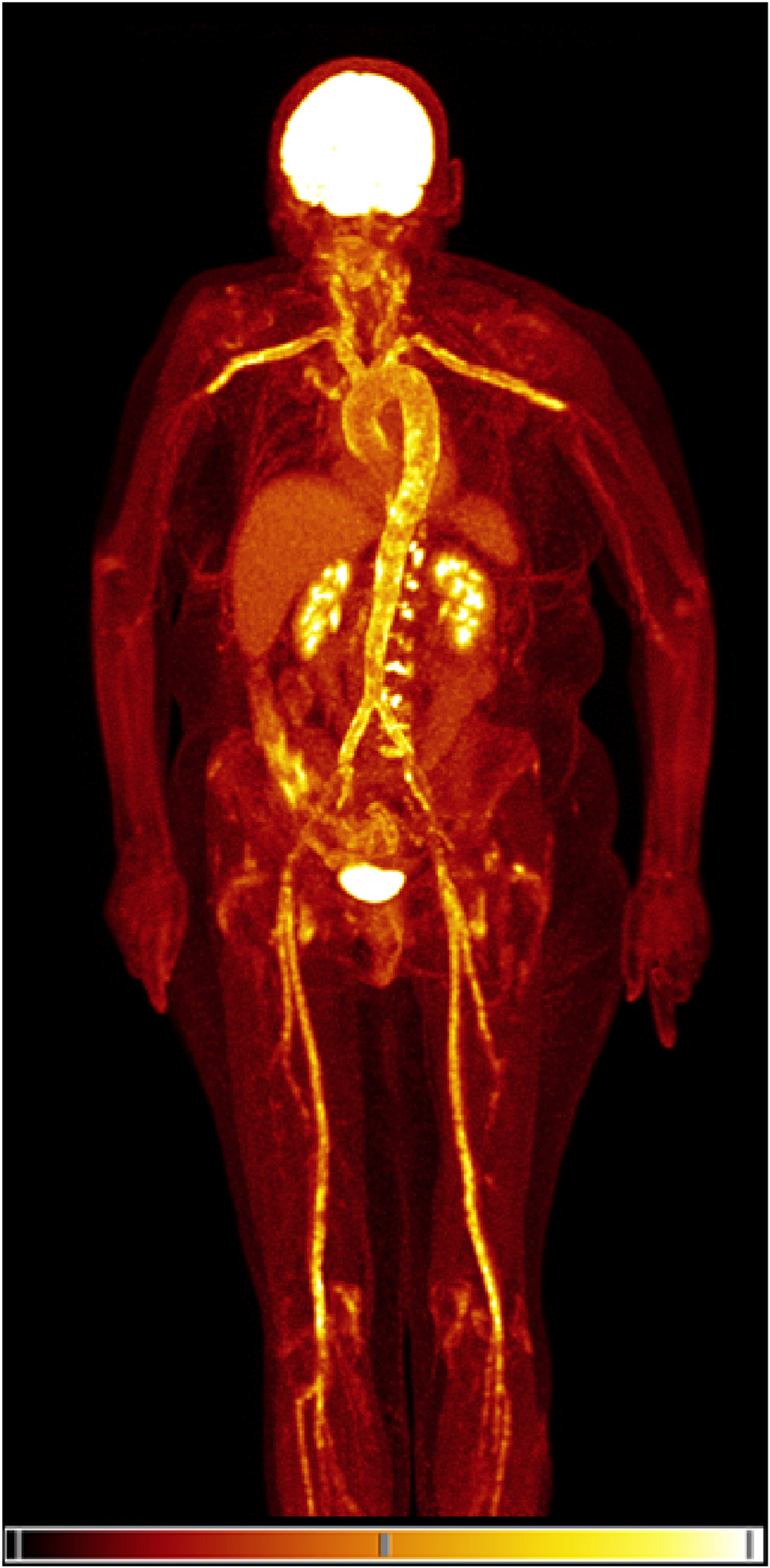
MIP View FDG-PET (Biograph Vision Quadra) scan in a 69-year-old female with lvGCA and PMR of the shoulders, hips, knees, and lumbar spine. MIP, maximum intensity projection; FDG-PET, [18F]-fluoro-2-deoxy-d-glucose positron emission tomography/computed tomography; lvGCA, large vessel GCA; PMR, polymyalgia rheumatica.

As a whole-body imaging technique, FDG-PET/CT visualizes increased metabolism in cells, thereby identifying neoplastic and inflammatory lesions. Intense and diffuse FDG uptake that involves the complete circumference of the vessel wall is characteristic of LVV (Figure 2) [28,29]. A recent meta-analysis showed an overall sensitivity of 80% and a specificity of 91% for FDG-PET/CT [30]. However, diagnostic accuracy studies vary in which arteries are being assessed. Whereas historically FDG-PET/CT could only reliably image lvGCA, recent studies in higher sensitive camera systems have shown that the presence of cGCA may also be established by FDG-PET/CT [31–33]. The European Alliance of Associations for Rheumatology (EULAR) recommends FDG-PET/CT as a second-line diagnostic imaging test after US. The most recent edition recommends assessment of the cranial arteries in addition to the large systemic arteries [34].

Whereas the hybrid imaging using low-dose CT as anatomical reference is well-established in GCA, hybrid systems using MRA as anatomical references have more recently become available. The potential benefits of such systems are clear, as they combine functional assessment with morphological inflammatory changes to the vessel wall, possibly working synergistically to improve diagnostic performance [35]. Additionally, MRA vessel wall thickening and enhancement do not always overlap with increased FDG-uptake in the same arterial segment [36,37]. As such, FDG-PET/MRA imaging may identify different stages of vessel wall inflammation and different stages within the LVV disease course [38,39]. However, prospective studies and studies including assessments of the cranial arteries using FDG-PET/MRA are still lacking.

FDG-PET/CT may also be used in cases of relapsing disease as disease signs, symptoms, and biochemical markers may be nonspecific [40]. However, FDG uptake may be present in patients with clinical remission, and therefore routine FDG-PET/CT imaging for follow-up purposes is not recommended in LVV patients [34]. However, high arterial FDG-uptake is related to an increased risk of later aneurysm formation and may therefore provide grounds for long-term monitoring of vessel wall damage [34,41].

It is important to realize that glucocorticoid treatment causes a significant decrease in FDG-uptake [42]. This complicates the use of FDG-PET/CT for diagnosis, as treatment is often started early, and in assessing disease activity during treatment. Moreover, atherosclerosis may cause low-grade FDG-uptake in the vessel wall, possibly mimicking vasculitis [28]; however, by looking carefully at the different FDG-uptake patterns it is possible to differentiate between LVV and atherosclerosis.

Adequate patient preparation for FDG-PET imaging is crucial to minimize physiologic tracer uptake in nontarget tissues such as the myocardium, skeletal muscle, urinary tract, and brown adipose tissue, while ensuring optimal uptake in diseased areas. This preparation involves fasting for at least 6 hours prior to FDG administration, though non-caloric beverages are permitted. Patients are advised to avoid strenuous physical activities for 24 hours before the scan and to relax in a temperature-controlled room immediately before and after the FDG injection to reduce muscle and brown fat uptake. Serum glucose levels are also a consideration, as levels above 7 mmol/L (126 mg/dL) can lead to suboptimal imaging by promoting FDG diversion to insulin receptor-rich organs [29].

## THE IMAGING MODALITY OF CHOICE

The choice of imaging modalities for assessing LVV depends on the clinical presentation. GCA is the most common form of LVV, and patients typically present with cranial symptoms and constitutional issues such as mild fever and weight loss. Therefore, US/CDUS of the head, neck, and upper extremities is recommended as the initial imaging modality [25]. Along with US/CDUS temporal artery biopsy can also be done; however, it has false-negative results due to the disease’s patchy nature. The diagnostic accuracy of the temporal artery biopsy is influenced by several factors, including the length of the specimen, with longer specimens generally providing higher diagnostic yields. The sensitivity of temporal artery biopsy was widely across studies but was estimated at 77% in a meta-analysis warranting cautious interpretation due to high heterogeneity between studies, while the utility of bilateral biopsies for increasing diagnostic yield is debated [43].

**Figure 3.**
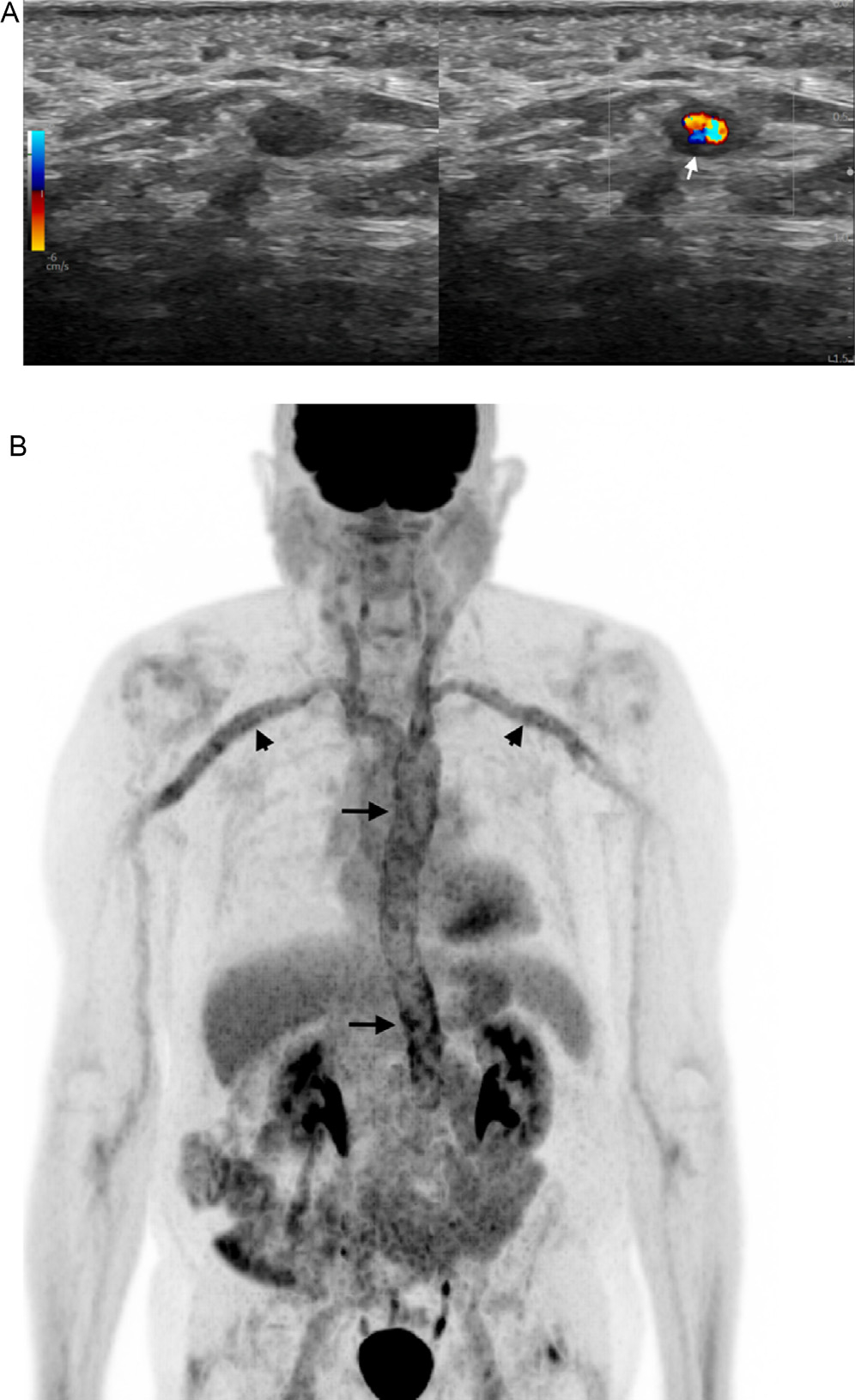
An 85-year-old male presented with fatigue elevated inflammatory markers, underwent US/CDUS of the head and neck (A), which demonstrated circumferential wall thickening (arrow), a classic halo sign of the temporal and supra-aortic arch vessels, including carotid and subclavian arteries, consistent with c-GCA. To assess aorta and the branches, the patient underwent FDG-PET/CT. MIP FDG-PET/CT image (B) showed intense FDG uptake throughout the aorta and supraaortic branches, which confirmed lvGCA. US/CDUS, Ultrasound with Color Doppler; cGCA, cranial GCA; FDG-PET/CT, [18F]-fluoro-2-deoxy-d-glucose positron emission tomography/computed tomography; MIP, maximum intensity projection; lvGCA, large vessel GCA.

**Figure 4.**
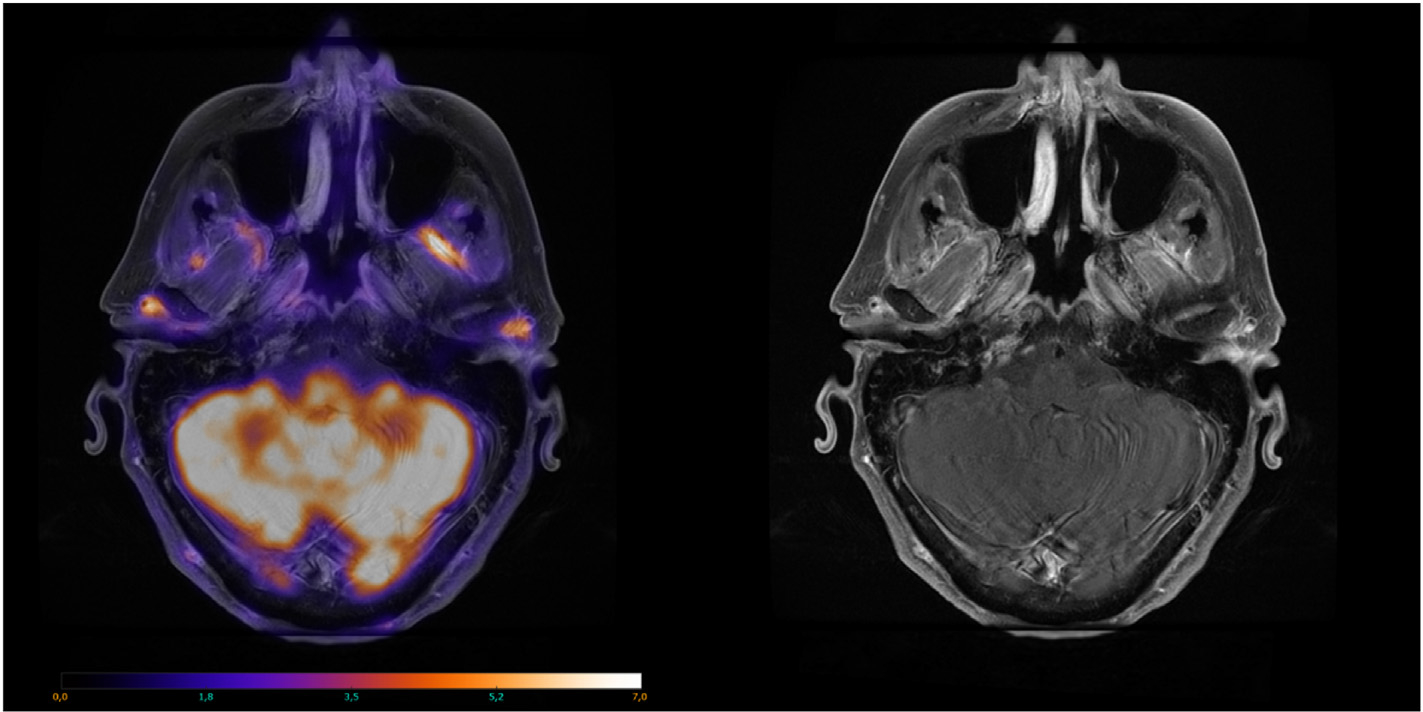
Left: Axial FDG-PET/MR image of a patient with cGCA. Increased uptake can be observed here in the common superficial temporal arteries, maxillary arteries, right posterior deep temporal artery, and right occipital artery. Right: Axial contrast-enhanced T1-weighted MR image in the same patient without FDG-PET overlay. Concentric vessel wall thickening and attenuation can be observed here in the right common superficial temporal artery and right occipital artery. Image courtesy of Lars Gormsen, MD, PhD, Aarhus University Hospital (Denmark). FDG-PET/MR, [18F]-fluoro-2-deoxy-d-glucose positron emission tomography/magnetic resonance; cGCA, cranial GCA.

In cases where US/CDUS fails to reveal vasculitis findings despite high clinical suspicion or as an additional diagnostic measure for confirming cGCA and assessing the aorta and its branches, FDG-PET/CT should be the next step (Figure 3). This whole-body approach aids in diagnosing LVV confined to the aorta, also identifying vasculitis mimickers like infection or malignancy, and offering functional data on vessels [44]. Additionally, combining anatomical imaging with CT angiography or MR angiography, based on institutional availability, holds synergistic potential for achieving an optimal diagnosis and enabling the assessment of vascular damage progression [29]. In addition to aortic imaging with FDG-PET/CT head and neck imaging should be performed to image superficial cranial arteries [34]. Similarly, correlative anatomical imaging with head MRA can be performed to assess superficial cranial arteries (Figure 4).

**Figure 5.**
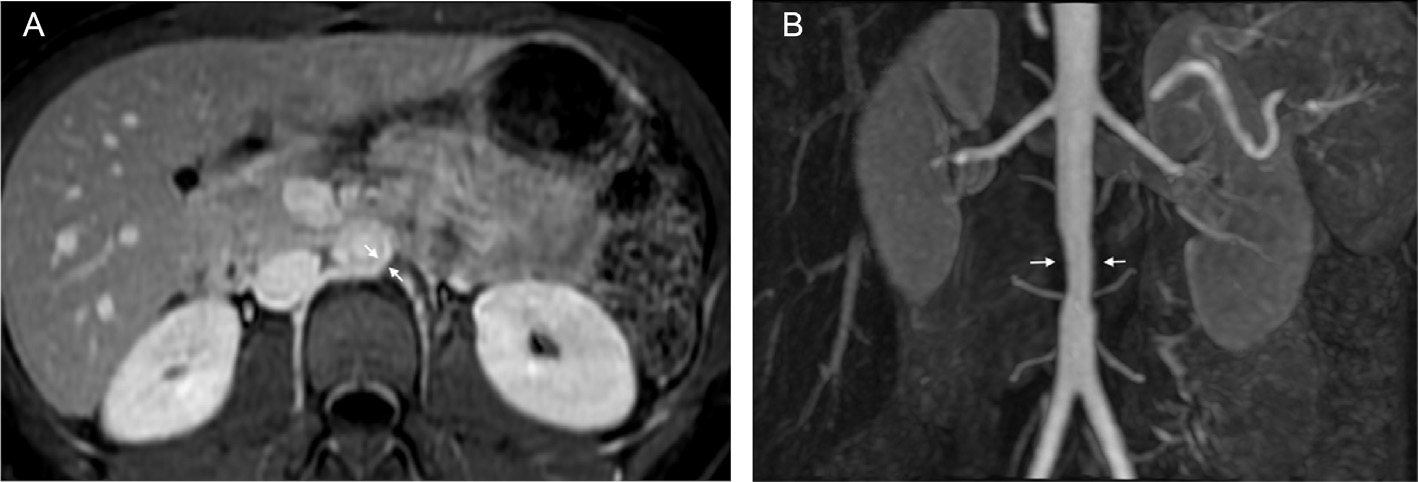
A 21-year-old female presented with dyspnea, chest pain, and elevated inflammatory markers. Given her young age, and suspected diagnosis of TAK, patient underwent MRA study. Axial image of the MRA of the abdomen (A) revealed segmental thickening (arrow) of the infrarenal abdominal aorta, with mild luminal narrowing (arrow) on the MIP MRA image (B). TAK, Takayasu’s arteritis; MRA, magnetic resonance angiography; MIP, maximum intensity projection.

**Figure 6.**
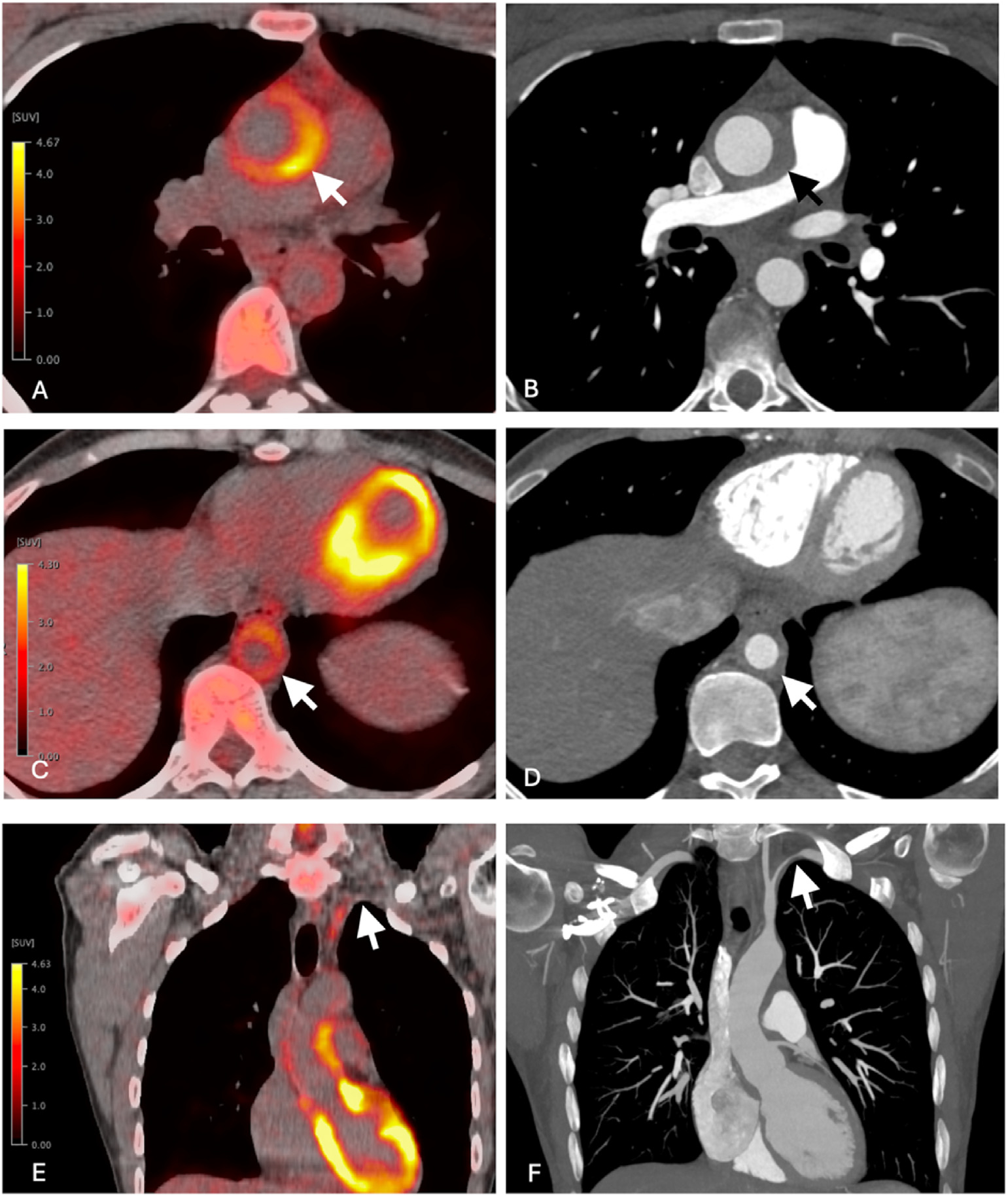
35-year-old female with a remote history of TAK presented with fatigue and elevated inflammatory markers. Given the expected chronic changes in the vessels, the decision has been made to proceed with FDG-PET/CTA to assess disease activity along with vascular complications. (A) Axial FDG-PET/CT image of the ascending aorta and (C) the descending aorta demonstrate circumferentially intense FDG uptake of the wall (arrows), corresponding to thickening (B,D) on axial CTA image (arrows). These findings are consistent with a relapse/active vasculitis. However, the coronal FDG-PET/CT image (E) of the chest, shows none-to-mild FDG uptake of the left subclavian artery (arrow), corresponding to severe narrowing (arrow) of the vessel on coronal MIP CTA image (F), most likely represents the sequela of prior vasculitis. TAK, Takayasu’s arteritis; FDG-PET/CTA, 18F]-fluoro-2-deoxy-d-glucose positron emission tomography/computed tomography angiography.

**Figure 7.**
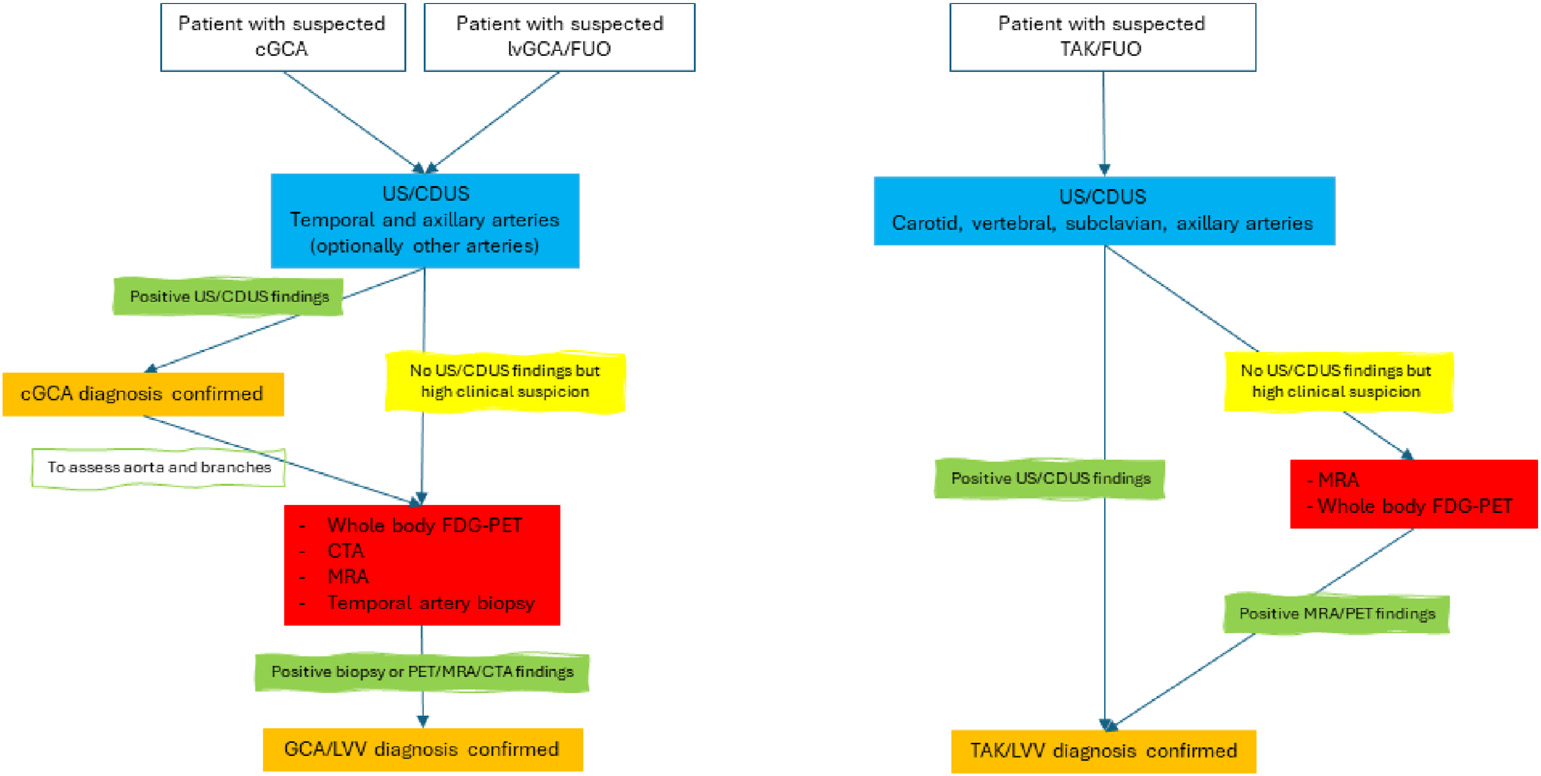
Algorithm for imaging technique selection in specific clinical situations according to recommendations [34].

In cases of suspected TAK in young women, after US/CDUS imaging of the neck and extremity vessels, MRA is recommended to minimize radiation exposure if the clinical suspicion is high (Figure 5). This is particularly crucial as these patients may require multiple imaging throughout their lifetime [25]. However, studies have suggested that FDG-PET/CT can offer additional insights into the local inflammatory features of vessels in TAK patients if needed (Figure 6) [45]. Similarly, a comprehensive prospective study comparing FDG-PET/CT with MRA in the diagnosis of LVV found that while MRA excels in evaluating anatomical changes and vascular damage, FDG-PET/CT provides more comprehensive information regarding disease activity [46]. Lastly, in patients with a clinical presentation of polymyalgia rheumatica (PMR) or fever of unknown origin, FDG-PET/CT is a preferred imaging modality for identifying concomitant PMR and alternative causes of inflammation. Figure 7 delineates the selection of imaging techniques in the most commonly encountered clinical situations based on recommendations.

## THE ROLE OF IMAGING IN THE FOLLOW-UP OF TREATMENT RESPONSE/MANAGEMENT

The role of imaging in monitoring treatment response and management is pivotal in LVV, essential for adjusting medications and addressing inherent vascular complications. However, literature and guideline recommendations on disease activity monitoring through imaging are currently limited [12].

Research using ultrasound (US) indicates that the halo sign observed in temporal arteries resolves within weeks to months of glucocorticoid treatment, whereas it persists in the larger arteries [47,48]. CTA studies reveal that although contrast enhancement diminishes in the aorta and its branches postglucocorticoid treatment vessel wall thickening persists in approximately two-thirds of patients [18]. In parallel with CTA, MRA studies also demonstrate a reduction in wall edema and enhancement, but wall thickening remains unchanged [49].

While FDG PET/CT is a valuable tool in LVV diagnosis, its role in disease monitoring remains unclear. Studies suggest that immunosuppressive treatment can decrease FDG uptake along the vessel wall; hence, FDG PET/CT is recommended within 3 days after initiating medication [42]. However, multiple investigations have identified persistent FDG uptake in the vessel wall among patients in clinical and biochemical remission, maybe due to vascular remodeling and/or smoldering LVV disease [40,50,51].

Although there have been important advances in follow-up imaging, the evidence is not strong enough to recommend imaging-based follow-up assessment of inflammation in all patients with LVV.

Nevertheless, multi-modality imaging may guide treatment decisions in selected cases where clinical evaluation alone is insufficient. Imaging for complications associated with LVV remains crucial, as discussed below.

## COMPLICATIONS IN LARGE VESSEL VASCULITIS

### Blindness

Sudden visual loss is a feared complication of cGCA. A meta-analysis reported that 24% of patients may develop blindness due to GCA [52]. In most cases, blindness develops due to anterior ischemic optic neuropathy (AION), caused by inflammation and occlusion of the posterior ciliar arteries, which are located downstream of the ophthalmic arteries [53]. The visual loss in GCA usually responds poorly to urgent methylprednisolone pulse therapy, which is mainly intended to protect the contralateral eye from developing blindness. Prodromic episodes of transient visual loss and diplopia may precede the occurrence of permanent visual loss. Rarely, visual loss in GCA is caused by posterior cerebral ischemia related to vertebral artery involvement. It is important to discriminate between AION developing due to GCA (arteritic AION) and other causes of AION, mainly atherosclerosis (nonarteritic AION). In the presence of other potential symptoms of GCA (e.g. headache, increased C-reactive protein, thrombocytosis), a full clinical workup with imaging and/or temporal artery biopsy is needed in patients presenting with AION. Specific ophthalmic imaging methods, such as fundus fluorescein angiography and optical coherence tomography, can provide important information regarding the presence of vascular defects in the eye [53,54]. MRI can be applied to demonstrate optic nerve inflammation and opthalmic artery involvement and to rule out alternative optic nerve pathologies causing visual loss [55,56].

### Atherosclerosis

Persistent inflammation in LVV, can lead to structural changes within the affected vessels, often resulting in accelerated atherosclerosis and subsequent vascular calcification. While this complication can occur in any form of LVV, substantial evidence demonstrated accelerated atherosclerosis particularly in patients with TAK [57,58]. The cardiovascular complications arising from such vascular changes contribute significantly to the morbidity and mortality in patients with LVV, depending on which vessels are involved. For instance, the involvement of the carotid arteries may lead to strokes. Similarly, GCA patients often succumb to complications related to accelerated atherosclerosis rather than from the vasculitis itself [59].

Results of a matched cross-sectional study indicated that patients with LVV have a higher prevalence of calcifications in the thoracic arteries compared to those without LVV [60]. However, this increased prevalence was not observed in the abdominal aorta or coronary arteries. Furthermore, in a comparison study on atherosclerotic calcifications between patients with LVV and those with hyperlipidemia, it was found that large vessel calcification was present in both groups [51]. Interestingly, in their study, there was a notably lower incidence of calcification in the coronary arteries in the LVV cohort. However, in a study performed in isolated TAK patients, coronary artery disease was found in more than half of the patents (53%) [24]. Therefore, young female patients diagnosed with LVV, especially those with TAK, presenting with chest pain, should be thoroughly evaluated for myocardial infarction.

For imaging, CTA is an effective method for assessing atherosclerosis. This modality can accurately quantify the extent of atherosclerosis in the major branches of the aorta, especially in the coronary artery branches. Although MRA can reveal vascular wall irregularities, suggesting atherosclerotic disease, visualizing calcification in the vessel wall can be challenging. In FDG-PET/CT, atherosclerosis can show FDG uptake, which may mimic active large vessel vasculitis (LVV). However, atherosclerosis typically presents with more focal uptake rather than diffuse. To address this diagnostic uncertainty, various methods of quantification have been proposed [28].

Given the increased risk of accelerated atherosclerosis in LVV, cardiovascular risk management is important to mitigate the risk and manage the cardiovascular aspects of the disease.

### Myocardial infarction/stroke

The cardiovascular (CV) disease burden in vasculitis is thought to be a result of the excess burden of a combination of the traditional CV risk factors and systemic inflammation, although the mechanism of inflammation differs between the vasculitides and remains understudied. Observational studies have reported a high prevalence of coronary artery disease in TAK with reports of CV events about 10 times more common than expected [61]. A large meta-analysis that included 35 studies and 3262 patients found a prevalence of myocardial infarction of 3.4% and 8.9% for stroke at any time during the disease course [62]. Coronary artery vasculitis prevalence varies widely between studies, predominantly due to the age evaluated, the lowest being in childhood onset TAK and higher rates observed in adult patients. Among TAK, autopsy data has revealed a prevalence of 11%–45% having coronary vasculitis. A study that examined TAK patients who underwent coronary angiography for symptoms or abnormal imaging findings found 54.6% (71/130) of patients having coronary involvement [63]. These findings have important implications for the detection and screening of TAK patients. Indeed, an important component of large vessel vasculitis assessment includes multimodality imaging that can aid in the diagnosis, monitoring, and management of the disease. Although invasive coronary angiography can rule out plaque rupture and dissection and assess complications of vasculitis, such as ostial lesions in TAK, whole body CT and coronary CT are useful in the less acute settings. In TAK, classic imaging features on coronary CTA are coronary artery ostial/proximal stenosis, which can be detected in up to >70% and followed by diffuse/focal distal narrowing or coronary artery aneurysms [64]. Assessment of peri-coronary adipose tissue density (PCAT) as a surrogate of systemic inflammation can also be utilized, with one study demonstrated a higher prevalence in TAK patients compared to patients with atherosclerotic coronary artery disease (CAD) [65].

### Microvascular dysfunction

**Figure 8.**
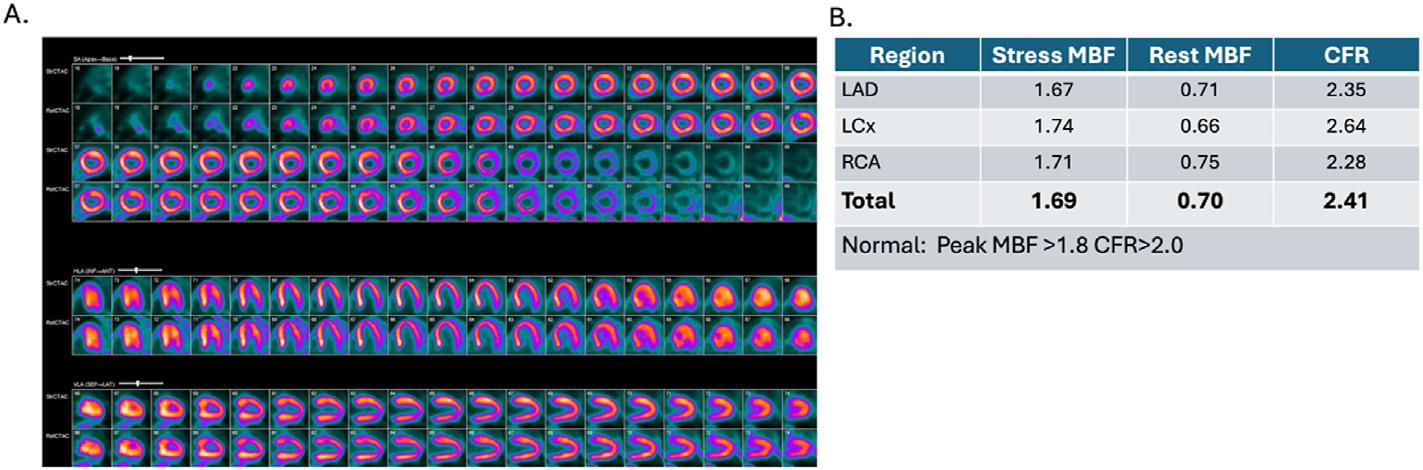
A 62-year old female with GCA in remission on immunosuppressive therapy with chest discomfort. Coronary microvascular dysfunction in a patient with GCA vasculitis in remission. A. ^13^N-ammonia perfusion PET. Perfusion images demonstrate the absence of stress-induced ischemia with vasodilator administration. Transmission CT scan revealed the absence of any coronary artery calcifications (not shown). B. Myocardial flow reserve demonstrates globally reduced peak myocardial blood flow (normal >1.8) with a preserved resultant MFR of 2.41. In the absence of flow limiting ischemia or any coronary artery calcification detected by CT, these results demonstrate diffuse mild coronary vasomotor dysfunction. GCA, giant cell arteritis; MFR, myocardial flow reserve; CT, computed tomography.

Coronary microvascular dysfunction has gained increasing recognition in patients with systemic inflammatory disorders [66–70]. Coronary microvascular function can be assessed through various different techniques, although each modality has pros and cons, including radiation, availability, and cost. Cardiac perfusion PET is the imaging modality that is the most well validated for the absolute quantification of myocardial blood flow and assessment of microvascular function, representing the gold standard. Other techniques include contrast echocardiography, Doppler transthoracic echocardiography, CT perfusion, and cardiac magnetic resonance (CMR) imaging [71]. Data on coronary vasomotor dysfunction in systemic vasculitis specifically are limited. Prior studies have demonstrated that brachial artery flow-mediated endothelium-dependent vasodilation is impaired in systemic vasculitis, which improves with immunosuppression therapy, although these studies did not specifically evaluate LVV [66,68]. A recent retrospective study, which specifically looked at LVV patients, demonstrated that coronary microvascular dysfunction detected by cardiac perfusion PET imaging was more prevalent among patients with vasculitis compared to age, gender, and risk factor matched individuals [72]. This study combined different types of vasculitides, with GCA being the most common (38%) type and a rare TAK rare incidence (12%). Moreover, 27% of the included patients did not have active vasculitis at the time of PET imaging. Patients with LVV had a prevalence of 38% of microvascular dysfunction (defined by a myocardial flow reserve (MFR) < 2) compared to 15% in controls, meaning that the coronary flow reserve (CFR) that was 19% lower in vasculitis cases (2.1 (.5) compared to 2.6 (.7), *P* = .003) (Figure 8). To understand the contribution of diffuse atherosclerosis, coronary artery calcium score was assessed. Approximately one third had no coronary artery calcium, with similar rates of burden atherosclerosis between vasculitis patients and controls, suggesting that coronary microvascular dysfunction (CMD) is not only a result of diffuse atherosclerosis and reflects coronary vasomotor dysfunction [62]. Other studies have similarly reported changes in the retinal vasculature among children with Kawasaki’s disease, reflecting another bed of microvascular dysfunction [73]. Unfortunately, limited data exists on the differences in microvascular function between the large, medium, and small vessel vasculitis or the relationship to disease severity within each of these disease states. Whether treatment with specific immunosuppressive therapy in GCA and TAK would lead to improvement in microvascular function is not known and is an important area for future investigation.

### Dilation/aneurysm and stenosis

**Figure 9.**
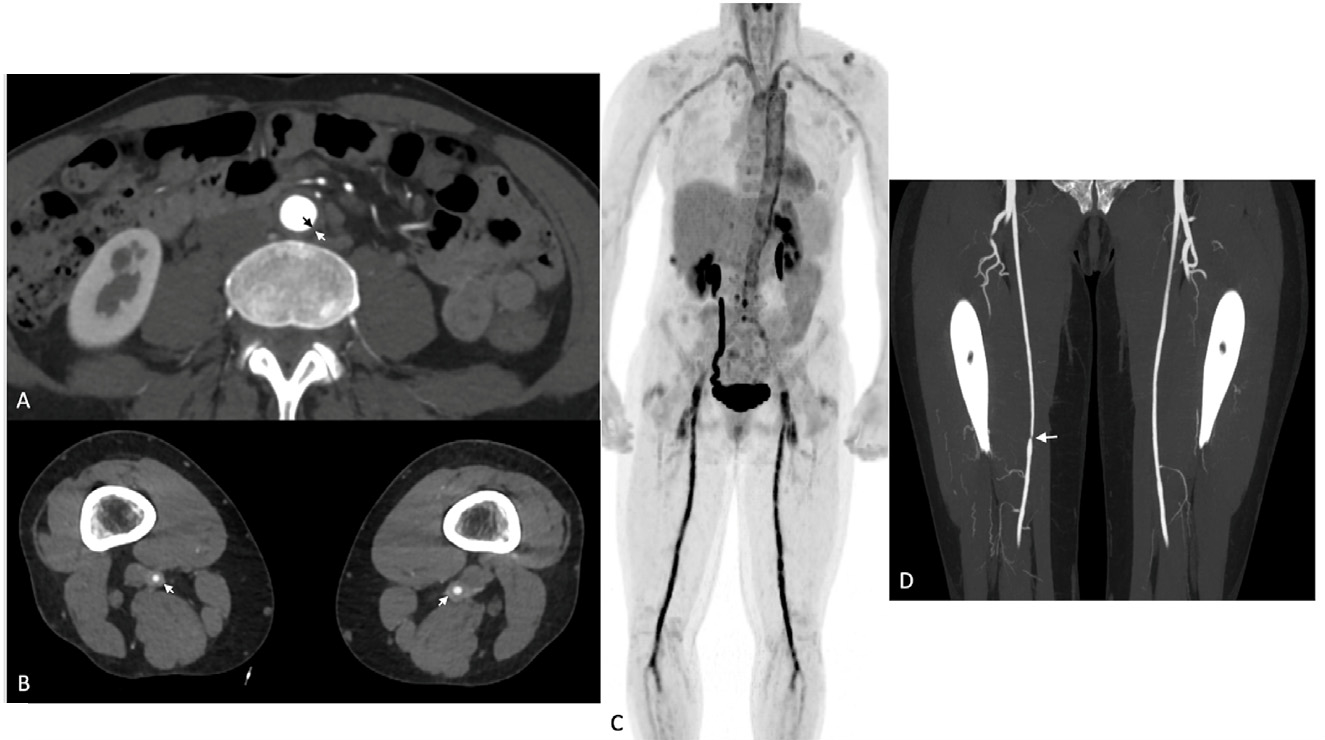
A 59-year-old female presented with claudication. Contrast-enhanced CTA of the abdomen/pelvis (Panel A) and lower extremities (Panel B) revealed circumferential wall thickening of the aorta and lower extremity arteries (arrows). Subsequent FDG-PET/CT imaging (Panel C) demonstrated moderate-to-intense FDG uptake in the corresponding vessels. A cranial ultrasound demonstrated the halo sign (not shown), and a subsequent temporal artery biopsy confirmed the diagnosis of GCA. Follow-up CTA after immunosuppressive therapy (Panel D) showed resolution of the wall thickening, but with severe stenosis of the distal superficial femoral artery (arrow). CTA, computed tomography angiography; FDG-PET/CT, [18F]-fluoro-2-deoxy-d-glucose positron emission tomography/computed tomography; GCA, giant cell arteritis.

**Figure 10.**
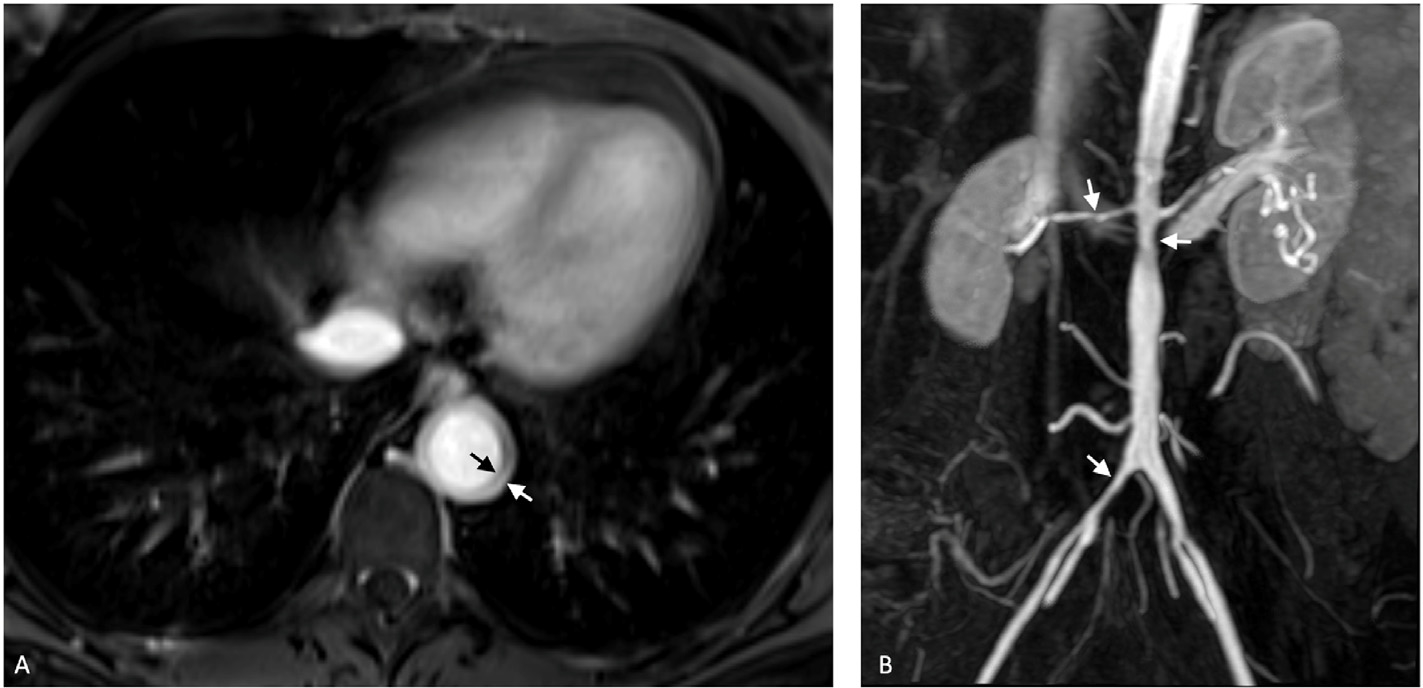
A 73-year-old female, with temporal artery biopsy-proven GCA, presented with chest pain. A postcontrast T1-weighted MRA image of the chest (A) demonstrates circumferential thickening of the descending thoracic aorta, consistent with lvGCA. A 45-year-old female with known TAK presented with hypertension. An abdominal MRA was performed. The MIP MRA image (B) of the abdomen reveals multifocal narrowing of the abdominal aorta, the right common iliac artery, and the right renal artery, as indicated by arrows. GCA, giant cell arteritis; MRA, magnetic resonance; angiography; TAK, Takayasu’s arteritis; MIP, maximum intensity projection.

Complications such as vessel aneurysm or stenosis occur at a higher rate in both GCA and TAK (Figures 9 and 10). However, there are some inherent differences. GCA commonly causes vessel dilatation/aneurysm, affecting up to 30% of patients [18,74]. A large study involving nearly 7000 GCA patients demonstrated that the risk of aortic aneurysm formation is two-fold higher in the GCA cohort compared to matched controls [75]. Although TAK can also cause aortic aneurysms, it is more frequently linked to arterial stenoses [76]. Depending on the arterial stenoses involved, patients with TAK can present with varying clinical symptoms, including neurological symptoms or subclavian steal syndrome due to involvement of the aortic arch branches, or hypertension due to renal artery stenoses [77].

US/CDUS is mostly utilized as an initial imaging tool to assess arterial complications related to the neck or extremities. However, in the presence of abnormal flow or imaging findings, additional imaging with either CTA or MRA should be considered to visualize the extent of the pathology. For thoracoabdominal vascular abnormalities, US/CDUS is usually limited; therefore, advanced imaging modalities are utilized. While both CTA and MRA can be used to assess these complications, MRA is the preferred modality in patients with TAK, particularly in younger individuals when they need multiple follow-ups [12].

### Multiorgan involvement

Although the outcome of LVV is relatively better compared to other inflammatory conditions, such as small or medium vessel vasculitides, it is important to recognize that LVV is not a benign entity. Its complications, often caused by the involvement of vessels supplying vital organs, can be life-threatening. The most common organ involvements include vision loss, stroke, and myocardial infarction, as detailed above. However, LVV can affect any organ in the body, including the lungs, kidneys, bowels, and extremities. While GCA more frequently causes vision loss or stroke, Takayasu arteritis TAK is commonly associated with a wider range of organ involvements [78]. In addition to head and neck involvement, GCA can affect extremities which may lead to limb claudication and a Raynaud-like phenomenon [31]. Severe stenosis of the pulmonary arteries, renal arteries, or mesenteric arteries can lead to corresponding organ involvement and are more frequently seen in patients with TAK [79]. A multicenter study on TAK involving 318 patients demonstrated that 20% of patients experienced strokes, 10% developed end-stage renal disease, and 6% suffered from heart failure, indicating higher rates of organ involvement [78]. One of the major organ involvements often overlooked in TAK is pulmonary artery/lung involvement. There is a higher incidence of patients in TAK developing pulmonary hypertension due to severe stenoses of pulmonary arteries [80]. Imaging modalities can be chosen based on the clinical presentation and the involved organs. While the head and neck, kidneys, lungs, and extremities can be assessed with both CTA and MRA, CTA is the ideal imaging modality for mesenteric/bowel assessment due to its superior spatial resolution. This allows for a detailed examination of distal small branches and bowel wall assessment.

## FUTURE TRENDS

Predictors for the development of thoracic aortic aneurysms have been reported with inconsistent results [5]. However, retrospective and prospective studies have consistently indicated that FDG uptake in large vessels at diagnosis increases the risk for aortic complications [41,81–84]. Higher FDG uptake is associated with a higher yearly increase in thoracic and aortic dimensions. Performing PET imaging at diagnosis may help estimate the risk for aortic aneurysm formation in the future. The mechanism of aneurysm formation in patients with GCA remains unclear. Some observers propose that persistent subclinical vasculitis leads to cumulative damage of the aortic wall [5,85]. There is no consensus on how to screen patients with GCA for the development of aortic aneurysms. Guidelines from the European Alliance of Associations for Rheumatology and the American College of Rheumatology are currently not available for screening aortic dilatation [34,86]. In contrast, the American Heart Association and the American College of Cardiology Joint Committee recommend annual imaging with CT, magnetic resonance imaging, or FDG-PET/CT(A) [87].

Developments in PET/CT camera systems, such as digital or total-body systems, may enhance sensitivity and spatial resolution with a better signal-to-noise ratio (i.e. vessel wall vs. lumen), including the possibility to scan at later time points while retaining adequate image quality [88]. Furthermore, these new-generation PET/CT scanners allow the administration of lower tracer activities to patients while achieving similar or even better image quality than conventional scanners. These systems, including the new-generation PET/MRI scanners, may also visualize pathologic uptake in smaller cranial vessels (e.g. temporal and vertebral arteries), aided by the application of artificial intelligence [89].

Combining total-body systems with more specific (immuno) or antifibroblast PET tracers for vasculitis would allow a more thorough insight into how specific cell subpopulations are involved and behave in the pathogenesis of vasculitis and the development of vascular complications [90]. Moreover, multiorgan changes and damages, and the kinetic uptake of specific PET tracers after appropriate treatment of vasculitis, could be assessed with total-body systems, something that was not possible before the development of this type of scanner. In imaging LVV, radiopharmaceuticals targeting specific cell populations may potentially be superior compared to the current reference FDG. Since persistent T cell and macrophage infiltration have been reported in the pathological process of LVV [91], imaging T cell and macrophage subsets could, in theory, be superior to FDG in the diagnostic imaging of GCA [92].

Photon-counting detector (PCD) CT represents a significant advancement in CT technology, demonstrating superior contrast-to-noise ratio, enhanced spatial resolution, and reduced calcium blooming artifacts compared to traditional energy-integrating detector (EID) CT. This technological leap is particularly beneficial in both qualitative and quantitative CT techniques. For instance, it enhances the assessment of vascular complications, including detailed evaluation of adipose tissue attenuation in coronary arteries. A recent study emphasizes the potential of PCD CT in accurately assessing epicardial adipose tissue attenuation and the fat attenuation index, illustrating its significant impact on evaluating vessel inflammation [93]. Diffusion-weighted imaging (DWI) is emerging as a promising MRI technique for detecting inflammation in large vessel vasculitis (LVV), aiding in distinguishing active from inactive disease. However, data on this technique is still limited [94]. A recent study has demonstrated that low b-value DWI outperforms T2-weighted imaging in detecting mural inflammation in patients with active Takayasu’s arteritis, suggesting its potential superiority in evaluating this condition [95].

The importance of an LVV-team in the diagnosis, management, and clinical outcomes of patients with LVV is of major importance. Imaging findings should always be used with clinical circumcision. Establishing multidisciplinary LVV-teams will result in an earlier and more accurate diagnosis of the primary disease and its complications, treatment, and optimized timing for intervention.

## CONCLUSIONS

The importance of vascular complications in LVV, and especially in TAK, is critical. Multimodality imaging, essential for diagnosing and identifying such complications depends on the clinical situation and disease stage. Techniques like US/CDUS, computed tomography angiography of the aorta (CTAA), MRA, and FDG-PET/CT, including emerging technologies like photon-counting detector CT, play a vital role. Additionally, new developments like novel-specific PET tracers show promise to enhance the precision and scope of imaging, thereby improving patient outcomes. Imaging is crucial in diagnosing LVV due to the difficulties of tissue biopsy. Despite advancements, particularly in FDG-PET/CT, there is a need for an improved imaging-based diagnostic algorithm and a better understanding of the most effective modality to assess treatment response. Further research is needed to explore these novel imaging techniques and tracers, especially in differentiating various vasculitides and assessing disease activity.

## Key points

- Large vessel vasculitis (LVV), including giant cell arteritis (GCA) and Takayasu’s arteritis (TAK), presents significant diagnostic and management challenges, with multimodality imaging playing a crucial role in diagnosis, monitoring treatment response and identifying vascular complications.
- Ultrasound with Color Doppler (US/CDUS) serves as a primary, noninvasive diagnostic tool, especially for GCA, due to its accessibility and cost-effectiveness.
- Advanced imaging techniques such as computed tomography angiography (CTA), magnetic resonance angiography (MRA), and [18F]-fluoro-2-deoxy-d-glucose positron emission tomography/computed tomography (FDG-PET/CT) provides detailed assessments of vessel wall thickness, inflammation, and complications like aneurysms or stenoses.
- Complications of LVV include blindness, atherosclerosis, myocardial infarction, stroke, and vessel aneurysms or stenosis, with imaging being crucial for early detection and management to prevent significant morbidity and mortality.
- The importance of a multidisciplinary approach (“LVV-team”) for the management of LVVis emphasized, integrating imaging findings with clinical judgment to optimize diagnosis, treatment, and monitoring, highlighting the evolving landscape of imaging technology in enhancing patient outcomes.

## ABBREVIATIONS

CTA: computed tomography angiography
DWI: diffusion-weighted imaging
EID: energy-integrating detector
GCA: giant cell arteritis
LVV: large vessel vasculitis
MFR: myocardial flow reserve
MIP: maximum intensity projection
MRA: magnetic resonance angiography
PET/CT: positron emission tomography/computed tomography
PMR: polymyalgia rheumatica
TAK: Takayasus arteritis
TOF: time-of-flight
US/CDUS: ultrasound with color Doppler

## References

[1] Jennette JC, Falk RJ, Bacon PA, Basu N, Cid MC, Ferrario F, 2012 revised International Chapel Hill Consensus Conference Nomenclature of Vasculitides. Arthritis Rheum 2013;65:1–11. 23045170 10.1002/art.37715

[2] van der Geest KSM, Sandovici M, van Sleen Y, Sanders JS, Bos NA, Abdulahad WH, Review: What Is the Current Evidence for Disease Subsets in Giant Cell Arteritis? Arthritis Rheumatol 2018;70:1366–76. 29648680 10.1002/art.40520 PMC6175064

[3] Serra R, Butrico L, Fugetto F, Chibireva MD, Malva A, De Caridi G, Updates in Pathophysiology, Diagnosis and Management of Takayasu Arteritis. Ann Vasc Surg 2016;35:210–25. 27238990 10.1016/j.avsg.2016.02.011

[4] Soriano A, Muratore F, Pipitone N, Boiardi L, Cimino L, Salvarani C. Visual loss and other cranial ischaemic complications in giant cell arteritis. Nat Rev Rheumatol 2017 Aug;13(8):476–84. doi: 10.1038/nrrheum.2017.98. Epub 2017 Jul 6. 28680132

[5] Mackie SL, Hensor EM, Morgan AW, Pease CT. Should I send my patient with previous giant cell arteritis for imaging of the thoracic aorta? A systematic literature review and meta-analysis. Ann Rheum Dis 2014;73:143–8. 23264356 10.1136/annrheumdis-2012-202145

[6] Ma LY, Li CL, Ma LL, Cui XM, Dai XM, Sun Y, Value of contrast-enhanced ultrasonography of the carotid artery for evaluating disease activity in Takayasu arteritis. Arthritis Res Ther 2019;21:24. 30651132 10.1186/s13075-019-1813-2 PMC6335720

[7] Schmidt WA, Kraft HE, Vorpahl K, Volker L, Gromnica-Ihle EJ. Color duplex ultrasonography in the diagnosis of temporal arteritis. N Engl J Med 1997;337:1336–42. 9358127 10.1056/NEJM199711063371902

[8] van der Geest KSM, Wolfe K, Borg F, Sebastian A, Kayani A, Tomelleri A, Ultrasonographic Halo Score in giant cell arteritis: association with intimal hyperplasia and ischaemic sight loss. Rheumatology (Oxford) 2021;60: 4361–6. 33355340 10.1093/rheumatology/keaa806 PMC8410002

[9] Schafer VS, Juche A, Ramiro S, Krause A, Schmidt WA. Ultrasound cut-off values for intima-media thickness of temporal, facial and axillary arteries in giant cell arteritis. Rheumatology (Oxford) 2017;56:1479–83. 28431106 10.1093/rheumatology/kex143

[10] Aschwanden M, Daikeler T, Kesten F, Baldi T, Benz D, Tyndall A, Temporal artery compression sign–a novel ultrasound finding for the diagnosis of giant cell arteritis. Ultraschall Med 2013;34:47–50. 22693039 10.1055/s-0032-1312821

[11] Maeda H, Handa N, Matsumoto M, Hougaku H, Ogawa S, Oku N, Carotid lesions detected by B-mode ultrasonography in Takayasu’s arteritis: “macaroni sign” as an indicator of the disease. Ultrasound Med Biol 1991;17:695–701. 1685816 10.1016/0301-5629(91)90101-2

[12] Duftner C, Dejaco C, Sepriano A, Falzon L, Schmidt WA, Ramiro S. Imaging in diagnosis, outcome prediction and monitoring of large vessel vasculitis: a systematic literature review and meta-analysis informing the EULAR recommendations. RMD Open 2018;4:e000612. 29531788 10.1136/rmdopen-2017-000612 PMC5845406

[13] Fernandez-Fernandez E, Monjo I, Peiteado D, Balsa A, De Miguel E. Validity of the EULAR recommendations on the use of ultrasound in the diagnosis of giant cell arteritis. RMD Open 2022;8. 10.1136/rmdopen-2021-002120 PMC8983999 35383122

[14] Sebastian A, Tomelleri A, Kayani A, Prieto-Pena D, Ranasinghe C, Dasgupta B. Probability-based algorithm using ultrasound and additional tests for suspected GCA in a fast-track clinic. RMD Open 2020;6. 10.1136/rmdopen-2020-001297 PMC7547539 32994361

[15] Sargi C, Ducharme-Benard S, Benard V, Meunier RS, Ross C, Makhzoum JP. Assessment and comparison of probability scores to predict giant cell arteritis. Clin Rheumatol 2023, Jan;43(1):357–65. 37525060 10.1007/s10067-023-06721-6 PMC10774184

[16] Barra L, Kanji T, Malette J, Pagnoux C, CanVasc. Imaging modalities for the diagnosis and disease activity assessment of Takayasu’s arteritis: a systematic review and meta-analysis. Autoimmun Rev 2018;17:175–87. 29313811 10.1016/j.autrev.2017.11.021

[17] Hartlage GR, Palios J, Barron BJ, Stillman AE, Bossone E, Clements SD, Lerakis S. Multimodality imaging of aortitis. JACC Cardiovasc Imaging 2014;7:605–19. 24925329 10.1016/j.jcmg.2014.04.002

[18] Prieto-Gonzalez S, Arguis P, Garcia-Martinez A, Espigol-Frigole G, Tavera-Bahillo I, Butjosa M, Large vessel involvement in biopsy-proven giant cell arteritis: prospective study in 40 newly diagnosed patients using CT angiography. Ann Rheum Dis 2012;71:1170–6. 22267328 10.1136/annrheumdis-2011-200865

[19] Ethiraj D, Kanase ND, Indiran V. Double ring sign in Takayasu arteritis. QJM 2020;113:764. 32073636 10.1093/qjmed/hcaa030

[20] Lariviere D, Benali K, Coustet B, Pasi N, Hyafil F, Klein I, Positron emission tomography and computed tomography angiography for the diagnosis of giant cell arteritis: A real-life prospective study. Medicine 2016;95:e4146. 27472684 10.1097/MD.0000000000004146 PMC5265821

[21] de Boysson H, Dumont A, Liozon E, Lambert M, Boutemy J, Maigne G, Giant-cell arteritis: concordance study between aortic CT angiography and FDG-PET/CT in detection of large-vessel involvement. Eur J Nucl Med Mol Imaging 2017;44:2274–9. 28736805 10.1007/s00259-017-3774-5

[22] Vaidyanathan S, Chattopadhyay A, Mackie SL, Scarsbrook AF. Comparative effectiveness of (18)F-FDG PET-CT and contrast-enhanced CT in the diagnosis of suspected large-vessel vasculitis. Br J Radiol 2018;91:20180247. 29927635 10.1259/bjr.20180247 PMC6223176

[23] Garcia-Martinez A, Arguis P, Prieto-Gonzalez S, Espigol-Frigole G, Alba MA, Butjosa M, Prospective long term follow-up of a cohort of patients with giant cell arteritis screened for aortic structural damage (aneurysm or dilatation). Ann Rheum Dis 2014;73:1826–32. 23873881 10.1136/annrheumdis-2013-203322

[24] Kang EJ, Kim SM, Choe YH, Lee GY, Lee KN, Kim DK. Takayasu arteritis: assessment of coronary arterial abnormalities with 128-section dual-source CT angiography of the coronary arteries and aorta. Radiology 2014;270:74–81. 24009351 10.1148/radiol.13122195

[25] Dejaco C, Ramiro S, Duftner C, Besson FL, Bley TA, Blockmans D, EULAR recommendations for the use of imaging in large vessel vasculitis in clinical practice. Ann Rheum Dis 2018;77:636–43. 29358285 10.1136/annrheumdis-2017-212649

[26] Adler S, Sprecher M, Wermelinger F, Klink T, Bonel H, Villiger PM. Diagnostic value of contrast-enhanced magnetic resonance angiography in large-vessel vasculitis. Swiss Med Wkly 2017;147:w14397. 28322426 10.4414/smw.2017.14397

[27] Rheaume M, Rebello R, Pagnoux C, Carette S, Clements-Baker M, Cohen-Hallaleh V, High-resolution magnetic resonance imaging of scalp arteries for the diagnosis of giant cell arteritis: results of a prospective cohort study. Arthritis Rheumatol 2017;69:161–8. 27483045 10.1002/art.39824

[28] Stellingwerff MD, Brouwer E, Lensen KJ, Rutgers A, Arends S, van der Geest KS, Different scoring methods of FDG PET/CT in giant cell arteritis: need for standardization. Medicine 2015;94:e1542. 26376404 10.1097/MD.0000000000001542 PMC4635818

[29] Slart R, Writing g, Reviewer g, Inflammation, Members of EC, Members of EI, FDG-PET/CT(A) imaging in large vessel vasculitis and polymyalgia rheumatica: joint procedural recommendation of the EANM, SNMMI, and the PET Interest Group (PIG), and endorsed by the ASNC. Eur J Nucl Med Mol Imaging 2018;45:1250–69. 29637252 10.1007/s00259-018-3973-8 PMC5954002

[30] Bosch P, Bond M, Dejaco C, Ponte C, Mackie SL, Falzon L, Imaging in diagnosis, monitoring and outcome prediction of large vessel vasculitis: a systematic literature review and meta-analysis informing the 2023 update of the EULAR recommendations. RMD Open 2023;9. 10.1136/rmdopen-2023-003379 PMC10450079 37620113

[31] Nielsen BD, Hansen IT, Kramer S, Haraldsen A, Hjorthaug K, Bogsrud TV, Simple dichotomous assessment of cranial artery inflammation by conventional 18F-FDG PET/CT shows high accuracy for the diagnosis of giant cell arteritis: a case-control study. Eur J Nucl Med Mol Imaging 2019;46:184–93. 30066157 10.1007/s00259-018-4106-0

[32] Sammel AM, Hsiao E, Schembri G, Bailey E, Nguyen K, Brewer J, Cranial and large vessel activity on positron emission tomography scan at diagnosis and 6 months in giant cell arteritis. Int J Rheum Dis 2020;23:582–8. 32100451 10.1111/1756-185X.13805

[33] Nienhuis PH, Sandovici M, Glaudemans AW, Slart RH, Brouwer E. Visual and semiquantitative assessment of cranial artery inflammation with FDG-PET/CT in giant cell arteritis. Semin Arthritis Rheum 2020;50:616–23. 32502725 10.1016/j.semarthrit.2020.04.002

[34] Dejaco C, Ramiro S, Bond M, Bosch P, Ponte C, Mackie SL, EULAR recommendations for the use of imaging in large vessel vasculitis in clinical practice: 2023 update. Ann Rheum Dis 2023. 10.1136/ard-2023-224543 37550004

[35] Einspieler I, Thurmel K, Pyka T, Eiber M, Wolfram S, Moog P, Imaging large vessel vasculitis with fully integrated PET/MRI: a pilot study. Eur J Nucl Med Mol Imaging 2015;42:1012–24. 25876704 10.1007/s00259-015-3007-8

[36] Laurent C, Ricard L, Fain O, Buvat I, Adedjouma A, Soussan M, Mekinian A. PET/MRI in large-vessel vasculitis: clinical value for diagnosis and assessment of disease activity. Sci Rep 2019;9:12388. 31455785 10.1038/s41598-019-48709-w PMC6711961

[37] Nienhuis P, Sørensen CM, Keller K, Hansen I, Naeser E, Nyhuus Bendix Rasch M, POS0700 metabolic and morphologic PET/MR features in cranial giant cell arteritis. Ann Rheum Dis 2023:635–6.

[38] Clemente G, Pereira RMR, Aikawa N, Silva CA, Campos LMA, Alves G, Is positron emission tomography/magnetic resonance imaging a reliable tool for detecting vascular activity in treated childhood-onset Takayasu’s arteritis? A multicentre study. Rheumatology (Oxford) 2022;61:554–62. 33718967 10.1093/rheumatology/keab255

[39] Cerne JW, Liu S, Umair M, Pathrose A, Moore JE, Allen BD, Combined modality PET/MR for the detection of severe large vessel vasculitis. Eur J Hybrid Imaging 2022;6:16. 35965266 10.1186/s41824-022-00136-3 PMC9376186

[40] van der Geest KSM, Treglia G, Glaudemans A, Brouwer E, Sandovici M, Jamar F, Diagnostic value of [18F]FDG-PET/CT for treatment monitoring in large vessel vasculitis: a systematic review and meta-analysis. Eur J Nucl Med Mol Imaging 2021;48:3886–902. 33942141 10.1007/s00259-021-05362-8 PMC8484162

[41] Moreel L, Coudyzer W, Boeckxstaens L, Betrains A, Molenberghs G, Vanderschueren S, Association between vascular (18)F-fluorodeoxyglucose uptake at diagnosis and change in aortic dimensions in giant cell arteritis: a cohort study. Ann Intern Med 2023;176:1321–9. 37782924 10.7326/M23-0679

[42] Nielsen BD, Gormsen LC, Hansen IT, Keller KK, Therkildsen P, Hauge EM. Three days of high-dose glucocorticoid treatment attenuates large-vessel 18F-FDG uptake in large-vessel giant cell arteritis but with a limited impact on diagnostic accuracy. Eur J Nucl Med Mol Imaging 2018;45:1119–28. 29671039 10.1007/s00259-018-4021-4

[43] Rubenstein E, Maldini C, Gonzalez-Chiappe S, Chevret S, Mahr A. Sensitivity of temporal artery biopsy in the diagnosis of giant cell arteritis: a systematic literature review and meta-analysis. Rheumatology (Oxford) 2020;59:1011–20. 31529073 10.1093/rheumatology/kez385

[44] Sammel AM, Hsiao E, Schembri G, Nguyen K, Brewer J, Schrieber L, Diagnostic Accuracy of Positron Emission Tomography/Computed Tomography of the Head, Neck, and Chest for Giant Cell Arteritis: A Prospective, Double-Blind, Cross-Sectional Study. Arthritis Rheumatol 2019. 10.1002/art.40864 30848549

[45] Incerti E, Tombetti E, Fallanca F, Baldissera EM, Alongi P, Tombolini E, (18)F-FDG PET reveals unique features of large vessel inflammation in patients with Takayasu’s arteritis. Eur J Nucl Med Mol Imaging 2017;44:1109–18. 28180963 10.1007/s00259-017-3639-y

[46] Quinn KA, Ahlman MA, Malayeri AA, Marko J, Civelek AC, Rosenblum JS, Comparison of magnetic resonance angiography and (18)F-fluorodeoxyglucose positron emission tomography in large-vessel vasculitis. Ann Rheum Dis 2018;77:1165–71. 29666047 10.1136/annrheumdis-2018-213102 PMC6045453

[47] Habib HM, Essa AA, Hassan AA. Color duplex ultrasonography of temporal arteries: role in diagnosis and follow-up of suspected cases of temporal arteritis. Clin Rheumatol 2012;31:231–7. 21743987 10.1007/s10067-011-1808-0

[48] Nielsen BD, Therkildsen P, Keller KK, Gormsen LC, Hansen IT, Hauge EM. Ultrasonography in the assessment of disease activity in cranial and large-vessel giant cell arteritis: a prospective follow-up study. Rheumatology (Oxford) 2023;62:3084–94. 36651670 10.1093/rheumatology/kead028

[49] Sun Y, Ma L, Ji Z, Zhang Z, Chen H, Liu H, Value of whole-body contrast-enhanced magnetic resonance angiography with vessel wall imaging in quantitative assessment of disease activity and follow-up examination in Takayasu’s arteritis. Clin Rheumatol 2016;35:685–93. 25665823 10.1007/s10067-015-2885-2

[50] Grayson PC, Alehashemi S, Bagheri AA, Civelek AC, Cupps TR, Kaplan MJ, 18 F-Fluorodeoxyglucose-Positron Emission Tomography As an Imaging Biomarker in a Prospective, Longitudinal Cohort of Patients With Large Vessel Vasculitis. Arthritis Rheumatol 2018;70:439–49. 29145713 10.1002/art.40379 PMC5882488

[51] de Boysson H, Aide N, Liozon E, Lambert M, Parienti JJ, Monteil J, Repetitive (18)F-FDG-PET/CT in patients with large-vessel giant-cell arteritis and controlled disease. Eur J Intern Med 2017;46:66–70. 28865740 10.1016/j.ejim.2017.08.013

[52] van der Geest KSM, Sandovici M, Brouwer E, Mackie SL. Diagnostic Accuracy of Symptoms, Physical Signs, and Laboratory Tests for Giant Cell Arteritis: A Systematic Review and Meta-analysis. JAMA Intern Med 2020;180:1295–304. 10.1001/jamainternmed.2020.3050 PMC7432275 32804186

[53] Vodopivec I, Rizzo JF 3rd Ophthalmic manifestations of giant cell arteritis. Rheumatology (Oxford) 2018;57:ii63–ii72. Vodopivec I, Rizzo JF, 3rd. Ophthalmic manifestations of giant cell arteritis. Rheumatology (Oxford) 2018;57:ii63–72. 29986083 10.1093/rheumatology/kex428

[54] Pichi F, Fragiotta S, Freund KB, Au A, Lembo A, Nucci P, Cilioretinal artery hypoperfusion and its association with paracentral acute middle maculopathy. Br J Ophthalmol 2019;103:1137–45. 30257961 10.1136/bjophthalmol-2018-312774

[55] D’Souza NM, Morgan ML, Almarzouqi SJ, Lee AG. Magnetic resonance imaging findings in giant cell arteritis. Eye (Lond) 2016;30:758–62. 26915748 10.1038/eye.2016.19 PMC4869132

[56] Geiger J, Ness T, Uhl M, Lagreze WA, Vaith P, Langer M, Involvement of the ophthalmic artery in giant cell arteritis visualized by 3T MRI. Rheumatology (Oxford) 2009;48:537–41. 19233887 10.1093/rheumatology/kep011

[57] Seyahi E, Ucgul A, Cebi Olgun D, Ugurlu S, Akman C, Tutar O, Aortic and coronary calcifications in Takayasu arteritis. Semin Arthritis Rheum 2013;43:96–104. 23351614 10.1016/j.semarthrit.2012.11.001

[58] Ucar AK, Ozdede A, Kayadibi Y, Adaletli I, Melikoglu M, Fresko I, Seyahi E. Increased arterial stiffness and accelerated atherosclerosis in Takayasu arteritis. Semin Arthritis Rheum 2023;60:152199. 37011578 10.1016/j.semarthrit.2023.152199

[59] Pugh D, Karabayas M, Basu N, Cid MC, Goel R, Goodyear CS, Large-vessel vasculitis. Nat Rev Dis Primers 2022;7:93. 34992251 10.1038/s41572-021-00327-5 PMC9115766

[60] Besutti G, Marvisi C, Mancuso P, Fari R, Monelli F, Revelli M, Prevalence and distribution of vascular calcifications at CT scan in patients with and without large vessel vasculitis: a matched cross-sectional study. RMD Open 2023;9. 10.1136/rmdopen-2023-003278 PMC10462964 37640517

[61] Clifford AH. Cardiovascular Disease in Large Vessel Vasculitis: Risks, Controversies, and Management Strategies. Rheum Dis Clin North Am 2023;49:81–96. 36424028 10.1016/j.rdc.2022.08.004

[62] Kim H, Barra L. Ischemic complications in Takayasu’s arteritis: A meta-analysis. Semin Arthritis Rheum 2018;47:900–6. 29198409 10.1016/j.semarthrit.2017.11.001

[63] Wang H, Liu Z, Shen Z, Fang L, Zhang S. Impact of coronary involvement on long-term outcomes in patients with Takayasu’s arteritis. Clin Exp Rheumatol 2020;38:1118–26. 32083549

[64] Kang E-J, Kim SM, Choe YH, Lee GY, Lee K-N, Kim D-K. Takayasu Arteritis: Assessment of Coronary Arterial Abnormalities with 128-Section Dual-Source CT Angiography of the Coronary Arteries and Aorta. Radiology 2014;270:74–81. 24009351 10.1148/radiol.13122195

[65] Wall C, Huang Y, Le EPV, Corovic A, Uy CP, Gopalan D, Pericoronary and periaortic adipose tissue density are associated with inflammatory disease activity in Takayasu arteritis and atherosclerosis. Eur Heart J Open 2021;1:oeab019. 34661196 10.1093/ehjopen/oeab019 PMC8508012

[66] Weber B, Biery DW, Singh A, Divakaran S, Berman AN, Wu WY, Association of inflammatory disease and long-term outcomes among young adults with myocardial infarction: the Mass General Brigham YOUNG-MI Registry. Eur J Prev Cardiol 2022;29:352–9. 33784740 10.1093/eurjpc/zwaa154 PMC8481343

[67] Weber BN, Stevens E, Barrett L, Bay C, Sinnette C, Brown JM, Coronary Microvascular Dysfunction in Systemic Lupus Erythematosus. J Am Heart Assoc 2021;10:e018555. 34132099 10.1161/JAHA.120.018555 PMC8403317

[68] Liao KP, Huang J, He Z, Cremone G, Lam E, Hainer JM, Coronary Microvascular Dysfunction in Rheumatoid Arthritis Compared to Diabetes Mellitus and Association With All-Cause Mortality. Arthritis Care Res (Hoboken) 2021;73:159–65. 31705724 10.1002/acr.24108 PMC7210065

[69] Amigues I, Russo C, Giles JT, Tugcu A, Weinberg R, Bokhari S, Bathon JM. Myocardial Microvascular Dysfunction in Rheumatoid Arthritis(Quantitation by (13) N-Ammonia Positron Emission Tomography/Computed Tomography). Circ Cardiovasc Imaging 2019;12:e007495. 30636512 10.1161/CIRCIMAGING.117.007495 PMC6361523

[70] Ishimori ML, Martin R, Berman DS, Goykhman P, Shaw LJ, Shufelt C, Myocardial ischemia in the absence of obstructive coronary artery disease in systemic lupus erythematosus. JACC Cardiovasc Imaging 2011;4:27–33. 21232700 10.1016/j.jcmg.2010.09.019

[71] Mathew RC, Bourque JM, Salerno M, Kramer CM. Cardiovascular Imaging Techniques to Assess Microvascular Dysfunction. JACC Cardiovasc Imaging 2020;13:1577–90. 31607665 10.1016/j.jcmg.2019.09.006 PMC7148179

[72] Weber B, Wallace ZS, Parks S, Cook C, Huck DM, Garshick M, Association Between Systemic Vasculitis and Coronary Microvascular Dysfunction in the Absence of Obstructive Coronary Artery Disease. Circ Cardiovasc Imaging 2023;16:e014940. 36649456 10.1161/CIRCIMAGING.122.014940 PMC9999265

[73] Lim EJ, Aris IM, Choo J, Wong TY, Li LJ. Association between Coronary Artery Measurements and Retinal Microvasculature in Children with New Onset of Kawasaki Disease. Sci Rep 2019;9:16714. 31723195 10.1038/s41598-019-53220-3 PMC6853953

[74] Nuenninghoff DM, Hunder GG, Christianson TJ, McClelland RL, Matteson EL. Incidence and predictors of large-artery complication (aortic aneurysm, aortic dissection, and/or large-artery stenosis) in patients with giant cell arteritis: a population-based study over 50 years. Arthritis Rheum 2003;48:3522–31. 14674004 10.1002/art.11353

[75] Robson JC, Kiran A, Maskell J, Hutchings A, Arden N, Dasgupta B, The relative risk of aortic aneurysm in patients with giant cell arteritis compared with the general population of the UK. Ann Rheum Dis 2015;74:129–35. 24095936 10.1136/annrheumdis-2013-204113

[76] Vautier M, Dupont A, de Boysson H, Comarmond C, Mirault T, Mekinian A, Prognosis of large vessel involvement in large vessel vasculitis. J Autoimmun 2020;108:102419. 32035747 10.1016/j.jaut.2020.102419

[77] Yang L, Zhang H, Jiang X, Song L, Qin F, Zou Y, Clinical Features and Outcomes of Takayasu Arteritis with Neurological Symptoms in China: A Retrospective Study. J Rheumatol 2015;42:1846–52. 26233498 10.3899/jrheum.150097

[78] Comarmond C, Biard L, Lambert M, Mekinian A, Ferfar Y, Kahn JE, Long-Term Outcomes and Prognostic Factors of Complications in Takayasu Arteritis: A Multicenter Study of 318 Patients. Circulation 2017;136:1114–22. 28701469 10.1161/CIRCULATIONAHA.116.027094

[79] Saadoun D, Vautier M, Cacoub P. Medium- and Large-Vessel Vasculitis. Circulation 2021;143:267–82. 33464968 10.1161/CIRCULATIONAHA.120.046657

[80] Gong J, Yang Y, Ma Z, Guo X, Wang J, Kuang T, Clinical and imaging manifestations of Takayasu’s arteritis with pulmonary hypertension: A retrospective cohort study in China. Int J Cardiol 2019;276:224–9. 30172475 10.1016/j.ijcard.2018.08.047

[81] Blockmans D, Coudyzer W, Vanderschueren S, Stroobants S, Loeckx D, Heye S, Relationship between fluorodeoxyglucose uptake in the large vessels and late aortic diameter in giant cell arteritis. Rheumatology (Oxford) 2008;47:1179–84. 18515868 10.1093/rheumatology/ken119

[82] de Boysson H, Liozon E, Lambert M, Parienti JJ, Artigues N, Geffray L, 18F-fluorodeoxyglucose positron emission tomography and the risk of subsequent aortic complications in giant-cell arteritis: A multicenter cohort of 130 patients. Medicine 2016;95:e3851. 27367985 10.1097/MD.0000000000003851 PMC4937899

[83] de Boysson H, Daumas A, Vautier M, Parienti JJ, Liozon E, Lambert M, Large-vessel involvement and aortic dilation in giant-cell arteritis. A multicenter study of 549 patients. Autoimmun Rev 2018;17:391–8. 29427822 10.1016/j.autrev.2017.11.029

[84] Muratore F, Crescentini F, Spaggiari L, Pazzola G, Casali M, Boiardi L, Aortic dilatation in patients with large vessel vasculitis: A longitudinal case control study using PET/CT. Semin Arthritis Rheum 2019;48:1074–82. 30424972 10.1016/j.semarthrit.2018.10.003

[85] Garcia-Martinez A, Hernandez-Rodriguez J, Arguis P, Paredes P, Segarra M, Lozano E, Development of aortic aneurysm/dilatation during the followup of patients with giant cell arteritis: a cross-sectional screening of fifty-four prospectively followed patients. Arthritis Rheum 2008;59:422–30. 18311764 10.1002/art.23315

[86] Hellmich B, Agueda A, Monti S, Buttgereit F, de Boysson H, Brouwer E, Update of the EULAR recommendations for the management of large vessel vasculitis. Ann Rheum Dis 2020;79:19–30. 31270110 10.1136/annrheumdis-2019-215672

[87] Writing Committee M, Isselbacher EM, Preventza O, Hamilton Black Iii J, Augoustides JG, Beck AW, 2022 ACC/AHA Guideline for the Diagnosis and Management of Aortic Disease: A Report of the American Heart Association/American College of Cardiology Joint Committee on Clinical Practice Guidelines. J Am Coll Cardiol 2022;80:e223–393. 36334952 10.1016/j.jacc.2022.08.004 PMC9860464

[88] Slart R, Tsoumpas C, Glaudemans A, Noordzij W, Willemsen ATM, Borra RJH, Long axial field of view PET scanners: a road map to implementation and new possibilities. Eur J Nucl Med Mol Imaging 2021;48:4236–45. 34136956 10.1007/s00259-021-05461-6 PMC8566640

[89] Duff LM, Scarsbrook AF, Ravikumar N, Frood R, van Praagh GD, Mackie SL, An Automated Method for Artifical Intelligence Assisted Diagnosis of Active Aortitis Using Radiomic Analysis of FDG PET-CT Images. Biomolecules 2023;13. 10.3390/biom13020343 PMC9953018 36830712

[90] van der Geest KSM, Sandovici M, Nienhuis PH, Slart R, Heeringa P, Brouwer E, Novel PET Imaging of Inflammatory Targets and Cells for the Diagnosis and Monitoring of Giant Cell Arteritis and Polymyalgia Rheumatica. Front Med (Lausanne) 2022;9:902155. 35733858 10.3389/fmed.2022.902155 PMC9207253

[91] Maleszewski JJ, Younge BR, Fritzlen JT, Hunder GG, Goronzy JJ, Warrington KJ, Clinical and pathological evolution of giant cell arteritis: a prospective study of follow-up temporal artery biopsies in 40 treated patients. Mod Pathol 2017;30:788–96. 28256573 10.1038/modpathol.2017.10 PMC5650068

[92] van der Geest KSM, Slijkhuis BGC, Tomelleri A, Gheysens O, Jiemy WF, Piccolo C, Positron Emission Tomography Imaging in Vasculitis. Cardiol Clin 2023;41:251–65. 37003681 10.1016/j.ccl.2023.01.012

[93] Mergen V, Ried E, Allmendinger T, Sartoretti T, Higashigaito K, Manka R, Epicardial Adipose Tissue Attenuation and Fat Attenuation Index: Phantom Study and In Vivo Measurements With Photon-Counting Detector CT. AJR Am J Roentgenol 2022;218:822–9. 34877869 10.2214/AJR.21.26930

[94] Ironi G, Tombetti E, Napolitano A, Campolongo M, Fallanca F, Incerti E, Diffusion-Weighted Magnetic Resonance Imaging Detects Vessel Wall Inflammation in Patients With Giant Cell Arteritis. JACC Cardiovasc Imaging 2018;11:1879–82. 30121275 10.1016/j.jcmg.2018.06.015

[95] Yang H, Lv P, Zhang R, Fu C, Lin J. Detection of mural inflammation with low b-value diffusion-weighted imaging in patients with active Takayasu Arteritis. Eur Radiol 2021;31:6666–75. 33569625 10.1007/s00330-021-07725-z

